# Increased serum level of soluble interleukin-2 receptor is associated with a worse response of metastatic clear cell renal cell carcinoma to interferon alpha and sequential VEGF-targeting therapy

**DOI:** 10.1186/s12885-017-3369-3

**Published:** 2017-05-25

**Authors:** Akinori Nukui, Akinori Masuda, Hideyuki Abe, Kyoko Arai, Ken-Ichiro Yoshida, Takao Kamai

**Affiliations:** Department of Urology, Dokkyo Medical University, 880 Kitakobayashi Mibu, Tochigi, 321-0293 Japan

**Keywords:** Soluble interleulin-2 receptor, Renal cell carcinoma, Interferon, Sorafenib, Axitinib

## Abstract

**Background:**

Renal cell carcinoma (RCC) is a tumor with immunogenic properties. Soluble interleukin-2 receptor (sIL-2R) has a role in T cell activation and may be important for immune regulation in various conditions, including infections, transplantation rejection, autoimmune inflammatory states, and cancer. We investigated the prognostic value of the serum sIL-2R level in patients with metastatic RCC receiving IFN-alpha and vascular endothelial growth factor (VEGF)-targeting therapy.

**Methods:**

We monitored the serum level of sIL-2R over time and examined phosphorylated Akt expression by the primary tumor in 47 patients with metastatic clear cell RCC (ccRCC) undergoing cytoreductive nephrectomy followed by first-line adjuvant therapy with IFN-alpha plus sequential VEGF-targeting therapy as second- or third-line adjuvant therapy.

**Results:**

A preoperative increase of the serum level of sIL-2R was correlated with a higher preoperative serum level of programmed cell death 1 (PD-1)-ligand 1 (PD-L1), increased expression of phosphorylated Akt by the primary tumor, and a worse response to IFN-alpha/sequential VEGF-targeting therapy, as well as being an independent prognostic factor for a shorter overall survival time by multivariate analysis. Over time, the serum sIL-2R level largely reflected the tumor response to therapy.

**Conclusions:**

Monitoring the serum level of sIL-2R may help to predict the biological behavior of ccRCC, its response to IFN-alpha/sequential VEGF-targeting therapy, and the prognosis.

## Background

The interactions between malignancies and the immune system of the host are extremely complex. Although the immune system theoretically suppresses tumor development and/or promotes tumor regression, it is currently accepted that it can also stimulate tumor growth. These opposing actions of the immune system have been summarized as cancer immunoediting (the three E’s: elimination, equilibrium, and escape) [1], and one of the “hallmarks of cancer” is the ability to evade host immunity [2]. Suppression of tumor development requires the generation and activation of tumor antigen–specific T cells, so activating the immune system to treat cancer via stimulation of T cells has long been an objective of studies on antitumor immunity. Multiple co-stimulatory receptors and negative regulators (or co-inhibitory receptors) interact to regulate the activation and proliferation of T cells, as well as the gain or loss of T cell effector function [3, 4].

Clear cell renal cell carcinoma (ccRCC) has the typical features of an immunogenic tumor, including numerous tumor-infiltrating T lymphocytes (TILs) and cytotoxic T cells, which identify and selectively destroy tumor cells, as well as circulating tumor-specific T cells [5, 6]. CD4-positive (CD4 + CD25 + Foxp3+) regulatory T cells (Tregs) play an essential role in immunosuppression and self-tolerance of tumor antigens in patients with cancer or tolerance of microbial antigens in patients with chronic infection [7]. It was reported that patients with metastatic RCC show an increase of CD4+ Tregs, while high-dose IL-2 significantly decreases CD4+ Tregs in patients with an objective response to this cytokine [8].

IL-2 is important for the growth and differentiation of both effector T (Teff) cells and CD4+ Tregs, thus promoting either immunostimulation or immunosuppression, and it not only has a critical role in protective immunity but also in peripheral immune tolerance mediated by CD4+ Tregs [9–11]. IL-2 must bind with the IL-2 receptor (IL-2R) on target cells to mediate these various actions. There are three subunits of the IL-2R: IL-2R alpha (CD25), IL-2R beta (CD122), and IL-2R gamma (CD132) [9–11]. T cells constitutively express IL-2R beta and IL-2R gamma, while expression of IL-2R alpha increases with T cell activation. Thus, IL-2R alpha can serve as a phenotypic marker for CD4+ Tregs [7]. IL-2R alpha is released by T cells as a soluble form (soluble interleukin-2 receptor: sIL-2R), and elevation of the sIL-2R level is detected in patients with infectious diseases, transplantation rejection, autoimmune inflammation, and cancer [9–11]. Thus, it seems that sIL-2R release promotes T cell activation and is important for immune regulation in various conditions.

IL-2R signaling regulates tolerance and immunity by inducing the transcription of target genes (such as CD25) via various signaling pathways, such as the Janus kinase (JAK)–signal transducer and activator of transcription (STAT) pathway; the phosphatidylinositol 3’kinase (PI3K), serine/threonine kinase Akt, and mammalian target of rapamycin (mTOR) pathway; and the mitogen-activated protein kinase (MAPK) pathway [9–11]. Among these pathways, there is marked activation of the PI3K/Akt/mTOR pathway in RCC [12]. Through cancer immunoediting, CD4+ Treg cells and programmed cell death 1 (PD-1)-ligand 1 (PD-L1) play an important role in promoting the escape phase of tumor growth in an immunosuppressive tumor microenvironment [1], while interaction between PD-1 and PD-L1 may be involved in the production of CD4+ Tregs [4]. In addition, aerobic glycolysis in tumor cells promotes depletion of extracellular glucose and leads to dysfunction of TILs, while expression of PD-L1 in tumor cells leads to constitutive activation of the Akt/mTOR pathway [13, 14]. Thus, sIL-2R could potentially be a biomarker for prediction of resistance and selection of therapy, but its role in human RCC has not been elucidated. Accordingly, we investigated serum sIL-2R, serum PD-L1, and phosphorylated Akt expression by the primary tumor in patients with metastatic ccRCC undergoing cytoreductive nephrectomy followed by IFN-alpha and sequential VEGF-targeting therapy. Our findings provide some insight into the clinical utility and biological significance of sIL-2R in ccRCC patients.

## Methods

### Patients

This was a retrospective study performed in 47 patients (32 men and 15 women) with histopathologically diagnosed metastatic ccRCC who underwent cytoreductive nephrectomy at our center between June 2007 and June 2014. Patients received cytoreductive nephrectomy before undergoing any other therapy. For staging of the tumor, all patients underwent preoperative CT and/or MRI. Postoperative follow-up ranged from 3 to 100 months, with a median of 31 months. Metastatic disease was evaluated by CT and/or MRI every 2 to 4 months. This study was conducted in accordance with the Declaration of Helsinki and was approved by the ethical review board of Dokkyo Medical University Hospital. Each patient signed a consent form that was approved by our institutional Committee on Human Rights in Research.

Enrollment criteria for this study were as follows: (1) age ≥ 18 years; (2) detection of metastatic disease at the time of cytoreductive nephrectomy for ccRCC; (3) first-line IFN-alpha therapy that was discontinued for medical reasons (e.g., progressive disease, stable disease, or intolerable adverse effects); (4) IFN-alpha plus low-dose sorafenib as second-line therapy with/without axitinib as third-line therapy (patients refractory to IFN-alpha plus sorafenib); and (5) available medical records for the entire period from the start of first-line therapy until final follow-up/death.

After cytoreductive nephrectomy, all 47 patients received adjuvant immunotherapy with IFN-alpha to treat their extra-renal disease. First-line therapy was provided with natural human IFN-alpha (3, 5, or 6 million units administered intravenously or intramuscularly 2–3 times weekly). Patients with refractory tumors (progressive disease: PD) received second-line therapy, which was IFN-alpha (3, 5, or 6 million units intravenously or intramuscularly 2–3 times weekly) combined with low-dose sorafenib (400 mg/day = 50% of the recommended starting dose) [15]. Since the recommended dose intensity of IFN-alpha and anti-VEGF agents is lower in Japan than in the USA or EU due to the smaller physique of Japanese patients, we administered a low dose of sorafenib to reduce toxicity and combined it with IFN-alpha to increase the antitumor activity [15, 16]. Some patients who showed a poor response to second-line therapy with IFN-alpha plus sorafenib subsequently received third-line therapy with axitinib (at the recommended starting dose of 10 mg/day). The attending physicians assessed tumor progression on the basis of imaging findings (enlargement of existing lesions or detection of new lesions), deterioration of the performance status, and exacerbation of symptoms such as cancer pain, fever, or weight loss. Dose reduction of IFN-alpha, sorafenib, and axitinib was performed for grade 3/4 toxicity. The response to treatment was assessed according to RECIST criteria [17]. Serum levels of sIL-2R (normal range: 135.0–483.0 U/ml) were measured every 1 to 3 months by LSI Medience Corporation (Tokyo, Japan), and the preoperative serum level of soluble PD-L1 was measured by using human PD-L1 (CSB-E13644h, Cusabio Biotech, Wuhan, China). The final follow-up date was determined by reviewing the medical records in October 2015.

### Western blotting

Samples from the resected primary tumors were subjected to western blotting, as reported previously [15, 18]. To compensate for variation in the expression of phosphorylated Akt (Ser-473) (pAkt(Ser-473)), tumor tissue samples and non-tumor tissue samples from the same patient were compared. The following antibodies were employed: a rabbit anti-human antibody targeting pAkt (Ser-473) (Cell Signaling Technology, Inc.; PhosphoPlus Akt (Ser-473) Antibody Kit; # 9270, Danvers, MA) and a beta-actin antibody (Millipore; # 1501R Bedford, MA).

### Statistical analysis

The Mann-Whitney *U* test was performed to compare two groups, while the Kruskal-Wallis test was employed for comparisons among at least three groups. Spearman’s rank correlation coefficient analysis was performed to assess the relationships between variables of interest. Cause-specific survival curves were created by the Kaplan-Meier method and differences between the curves were assessed with the log-rank test. The impact on survival of the preoperative sIL-2R level, preoperative PD-L1, pAkt(Ser-473), histological grade, pT stage, pN stage, and microscopic vascular invasion was investigated by univariate and multivariate Cox proportional hazards analysis. In all analyses, *P* < 0.05 indicated statistical significance. Analyses were done with commercial software [18].

## Results

The clinical characteristics and outcomes of the patients are summarized in Table 1.

**Table 1:** Background of 47 metastatic clear cell RCCs

		1st line	2nd line (*n* = 41)		3rd line (*n* = 20)	
		IFN-alpha	IFN-alpha + sorafenib		Axitinib	
		CR/PR/SD > 24w (*n* = 6)	CR/PR/SD > 24w (*n* = 19)	SD < 24w/PD (*n* = 22)	CR/PR/SD > 24w (*n* = 9)	SD < 24w/PD (*n* = 11)
Sex (Male / Female)	32 / 15					
Years (median)	39–78 (65)					
ECOG PS^a^ (0 / 1 / 2)	29 / 14 / 4	6 / 0 / 0	13 / 6 / 0	10 / 8 / 4	4 / 5 / 0	2 / 8 / 1
MSKCC^a^ (Fav / Int / Poor)	24 / 15 / 8	6 / 0 / 0	9 / 7 / 0	9 / 8 / 8	2 / 7 / 0	1 / 8 / 2
Duration of IFN-alpha^a^ (mean: months)		7–46 (15.9)				
Duration of pre-IFN-alpha^a^ (mean: months)			1–31 (7.4)			
Duration of IFN-alpha + sorafenib (mean: months)			1–81 (19.7)			
Duration of axitinib (mean: months)					1–37 (11.6)	
Metastatic lesions^a^ (numbers)						
PUL	46	6	18	22	9	11
PLE	6	1	2	3	2	2
HEP	7	0	3	4	2	2
OSS^a^	11	0	6	5	3	3
LYM	12	0	6	6	3	4
Others	3	0	0	3	0	2

ECOG PS^a^: Eastern Cooperative Oncology Group (ECOG) performance status

MSKCC^a^: Memorial Sloen-Kettering Cancer Center, *Fav* Favorable, *Int* Intermediated, *Poor* Poor risk

Duration of IFN-alpha^a^: Duration of IFN-alpha monotherapy

Duration of pre-IFN-alpha^a^: Duration of IFN-alpha monotherapy prior toIFN-alpha plus sorafenib

Metastatic lesions^a^; *PUL* Lung, *PLE* Pleura, *HEP* Liver, *OSS* Bone, *LYM* lymph node

OSS^a^: Treatment option with Radiation plus Bisphosphonate or Denosmab

The preoperative serum sIL-2R level ranged from 114.2 to 2200.9 U/ml (mean ± S.D. = 601.5 ± 503.8 U/ml). None of the patients had inflammatory and/or autoimmune diseases, so preoperative sIL-2R levels exceeding the median value (498.8 U/ml) were not derived from concomitant diseases.

An increase of the preoperative sIL-2R level was detected in patients with poorly differentiated cancer (Fuhrman grade 1/2; mean ± S.D. = 322.9 ± 264.6, Fuhrman grade 3/4; 778.8 ± 594.5, *P* = 0.002), local invasion (pT1/2; mean ± S.D. = 230.7 ± 111.5, pT3/4; 667.9 ± 556.9, *P* = 0.0146), lymph node metastasis (pN0; mean ± S.D. = 490.0 ± 506.7, pN1/2; 834.6 ± 543.3, *P* = 0.0143), and vascular invasion (negative; mean ± S.D. = 269.0 ± 217.6, positive; 673.3 ± 560.4, *P* = 0.0276).

Among 47 patients who had metastasis when they underwent cytoreductive nephrectomy and received IFN-alpha as first-line adjuvant therapy, six patients showed a complete response (CR), partial response (PR), or stable disease (SD) for >24 weeks, while progression occurred in the other 41 patients and they were given IFN-alpha combined with low-dose sorafenib as second-line therapy. When evaluated from the best response, 19 of these 41 patients displayed a good response to IFN-alpha plus sorafenib, while the other 22 patients did not respond. Eight of the 19 responders eventually became resistant to second-line therapy. Ten of the 22 non-responders subsequently received best supportive care. Among the 20 patients (12/22 non-responders and 8/19 responders to second-line therapy) who received axitinib as third-line therapy, nine patients (4/12 non-responders and 5/8 responders to second-line therapy) showed a good response, while the remaining 11 patients were non-responders.

### Preoperative sIL-2R level and response of metastatic ccRCC

A lower preoperative serum sIL-2R level showed a correlation with a good response (complete response, partial response, or stable disease for >24 weeks) to either IFN-alpha monotherapy, IFN-alpha plus sorafenib, or axitinib (Table 2). When the patients displaying a good response to IFN-alpha (*n* = 6), IFN-alpha plus sorafenib (*n* = 19), or axitinib (4/12 non-responders to second-line therapy) were combined in a good response group (*n* = 29), the preoperative serum sIL-2R level was lower in this group compared with the group showing a poor response to any of these agents (i.e., stable disease for <24 weeks or progressive disease) (*P* = 0.0046, Table 2).

**Table 2:** Relationship between molecules and treatment outcome

	sIL-2R	*p* value	PD-L1	*p* value	pAkt (Ser-473)	*p* value
	(U/ml)		(pg/ml)			
	mean ± S.D		mean ± S.D		mean ± S.D	
IFN-alpha group						
IFN alone: CR/PR/SD > 24w* (*n* = 6)	123.9 ± 43.1	0.01	17.3 ± 13.0	0.02	2.67 ± 0.93	0.01
IFN + Sor: CR/PR/SD > 24w* (*n* = 19)	331.6 ± 207.7		18.6 ± 15.8		3.93 ± 2.09	
IFN + Sor: SD < 24w/PD* (*n* = 22)	567.3 ± 577.6		60.1 ± 98.2		6.42 ± 2.52	
Axitinib: CR/PR/SD > 24w* (*n* = 9)	433.3 ± 205.2		27.2 ± 16.4		5.95 ± 2.85	
Axitinib: SD < 24w/PD* (*n* = 11)	1050.1 ± 710.8		43.8 ± 24.6		7.01 ± 2.57	
1st and/or 2nd and/or 3rd therapy*						
CR/PR/SD > 24w* (*n* = 29)	367.1 ± 242.3	0	18.0 ± 12.9	0	3.69 ± 1.97	0
SD < 24w/PD* (*n* = 18)	784.5 ± 558.4		41.6 ± 20.7		6.67 ± 2.51	

CR/PR/SD>24w* : complete, partial, or stable with > 24 weeks response

SD<24w/PD* : stable disease for < 24 weeks or progressive disease

1st and/or 2nd and/or 3rd therapy* : IFN-alpha (1st-line), IFN-alpha + Sorafenib (2nd-line), Axitinib (3rd-line)

**Fig. 1:**
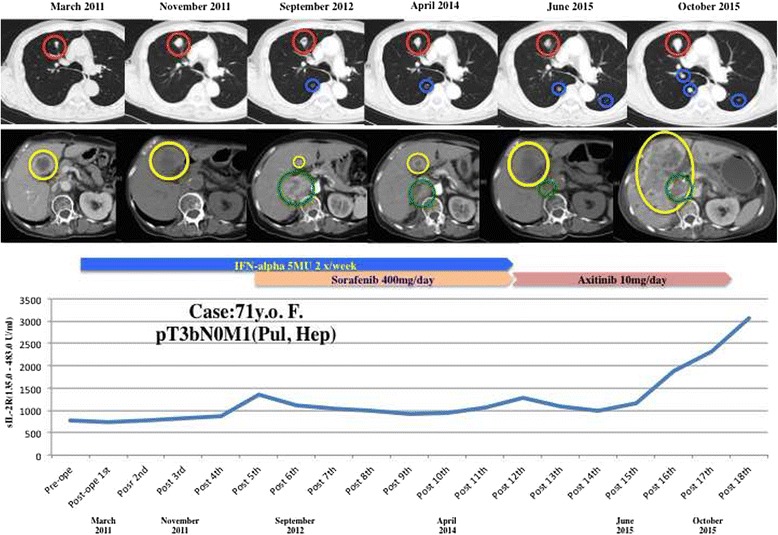
Disease status and the serum level of soluble interleukin-2 receptor (sIL-2R). The serum level of sIL-2R (normal range: 135.0–483.0 U/ml) was measured before cytoreductive nephrectomy (pre-ope) and every 1 to 3 months after nephrectomy (post-ope). A patient with metastatic RCC arising in the right kidney underwent cytoreductive right nephrectomy, and then received adjuvant immunotherapy with IFN-alpha (5 million units intramuscularly twice a week) as first-line therapy for extra-renal disease for 10 months. Both lung metastases (*red* and *blue cycles*) and liver metastases (*yellow cycles*) showed gradual progression during IFN-alpha treatment. When new retroperitoneal lesions (*green cycles*) appeared and sIL-2R began to increase, the patient received concomitant treatment with IFN-alpha (5 million units intramuscularly twice a week) and low-dose sorafenib (400 mg/day; half of the recommended starting dose of 800 mg/day) for 22 months as second-line therapy. The liver and retroperitoneal metastases gradually became smaller while sIL-2R was stable. After sIL-2R began to rise again and metastatic liver lesions began to enlarge, this patient subsequently received axitinib (recommended starting dose of 10 mg/day) as third-line therapy. The sIL-2R level and liver metastases remained stable for over 10 months, but sIL-2R began to rise rapidly again and the liver lesions rapidly progressed, then after which the patient died

**Fig. 2:**
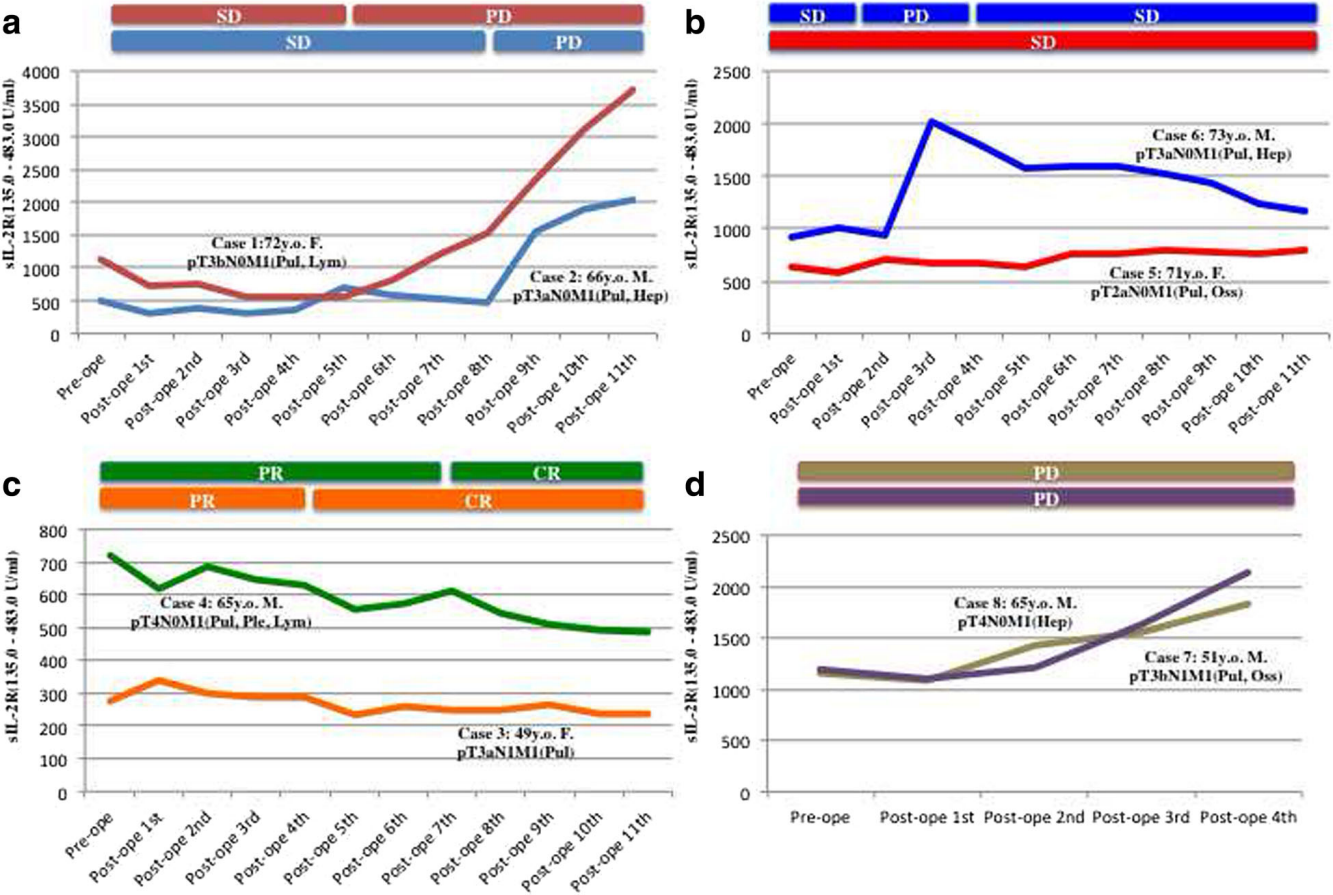
Clinical course and changes of the serum level of soluble interleukin-2 receptor (sIL-2R). The serum sIL-2R level (normal range: 135.0–483.0 U/ml) was measured before cytoreductive nephrectomy (pre-op) and every 1 to 3 months after nephrectomy (post-op). **a** Two patients showed a response to first-line and/or second-line therapy, but then gradually developed resistance along with elevation of sIL-2R. Although they subsequently received second-line or third-line therapy, respectively, they did not respond and sIL-2R continued to increase until death. **b** Two patients had relatively long-term stable disease while receiving first-line to third-line therapy. In these patients, sIL-2R remained stable or decreased gradually over time. **c** Two patients showed a good response to first-line, second-line, or third-line therapy. In these patients, the sIL-2R level generally remained within the normal range or decreased toward the normal range. **d** Two patients had a poor response to first-line and second-line therapy. In both patients, the sIL-2R level initially elevated and continued to rise further until death

Analysis of the time course of serum sIL-2R levels revealed that they largely paralleled the response to therapy (Fig. 1). For example, serum sIL-2R began to increase if a patient had a poor response to therapy and continued to increase thereafter (Fig. 2a), sIL-2R remained stable (even if it was high) or decreased gradually in patients with relatively long-term stable disease (Fig. 2b), sIL-2R was stable within the normal range or decreased toward normal in patients with a good response to any of the agents (Fig. 2c), or sIL-2R remained high and continued to rise further in patients responding poorly to any agent (Fig. 2d).

Elevated pAkt(Ser-473) expression in the primary tumor was found to show a correlation with a poor response to IFN-alpha and sequential VEGF-targeting therapy (*P* = 0.0021, Table 2).

**Fig. 3:**
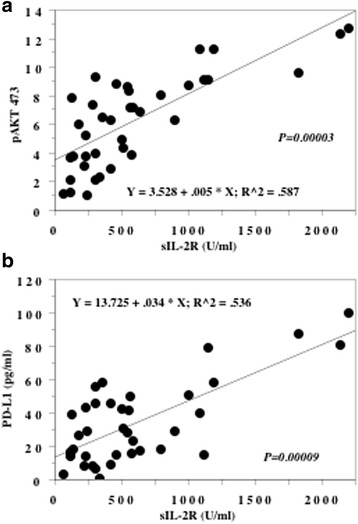
Spearman rank correlation between sIL-2R and Akt in the primary tumors and PD-L1. Spearman rank correlation between the preoperative serum sIL-2R level and the expression levels of phosphorylated Akt(Ser-473) in the primary tumors (**a**), and preoperative serum PD-L1 level (**b**)

When the relation between the preoperative serum sIL-2R level and pAkt(Ser-473) expression by the primary tumor was investigated, sIL-2R was positively correlated with pAkt(Ser-473) (r^2^ = 0.59, *P* = 0.00003, Fig. 3a).

Elevation of the preoperative serum level of PL-L1 was also found to be associated with a poor response to IFN-alpha and sequential VEGF-targeting therapy (*P* = 0.00004, Table 2), while preoperative sIL-2R and PD-L1 levels demonstrated a positive correlation (r^2^ = 0.54, *P* = 0.00009, Fig. 3b).

### Association of the serum sIL-2R level with overall survival

**Fig. 4:**
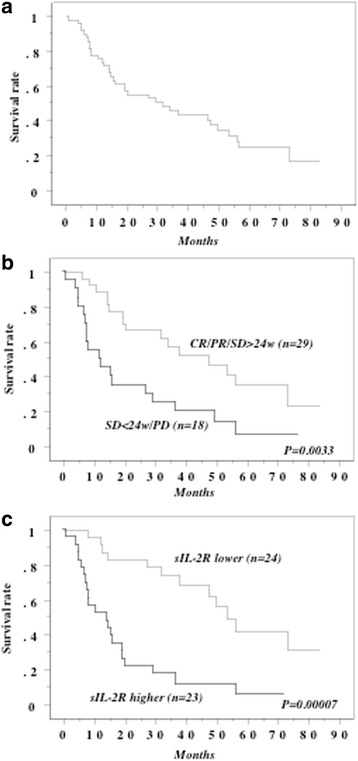
Overall survival curve in all patients. **a** Overall survival curve in all patients. **b** The patients with better response either for IFN-alpha, IFN-alpha plus sorafenib, or axitinib showed longer survival than those with poorer response. **c** This survival curve is based on the median values of preoperative serum sIL-2R level in all cases. The cases were divided into two groups at this level - high and low value. *P* value was analyzed by log-rank test

The median overall survival time (OS) of all patients after cytoreductive nephrectomy and IFN-alpha therapy was 31.9 months (Fig. 4a).

In patients with a favorable response to either IFN-alpha as first-line therapy, IFN-alpha plus low-dose sorafenib as second-line therapy, or axitinib as third-line therapy, median OS was 47.2 months, while median OS was only 11.9 months for patients responding poorly to any agent (*P* = 0.0033, Fig. 4b).

When the median preoperative serum level of sIL-2R (498.8 U/ml) was employed as the cut-off value to divide the patients into two groups, a higher sIL-2R level and shorter overall survival were associated according to Kaplan-Meier analysis (*P* = 0.00007, Fig. 4c).

A higher serum sIL-2R level (median: 498.8), higher serum PD-L1 level (median: 27.0), higher pAkt(Ser-473) expression (median: 5.63), undifferentiated tumor histology, and regional lymph node metastasis were all associated with shorter overall survival according to Cox univariate analysis, but only sIL-2R and PD-L1 were confirmed to have an impact by multivariate analysis (Table 3).

**Table 3:** Cox regression analysis for various potential prognostic factors in overall survival

Variable	Unfavorable/ favorable characteristics	No. of patients	Univariate (U)			Multivariate (M)		
			Relative risk	95% confidential interval	*P* value	Relative risk	95% confidential interval	*P* value
sIL-2R	high / low	23 / 24	4.44	2.094–9.426	0	2.62	1.161–6.452	0.0167
PD-L1	high / low	23 / 24	3.980	1.891–8.378	0	3.89	1.389–10.898	0.0097
pAkt	high / low	23 / 24	2.91	1.189–7.137	0.02	1.59	0.651–2.326	0.4539
Grade	4 / 3 / 2 / 1	4 / 21 / 19 / 3	3.105	1.711–5.634	0	1.87	0.974–3.588	0.06
pT	4, 3 / 2, 1	38 / 9	2.04	0.766–5.411	0.15			
pN	2,1 / 0	13 / 34	2.79	1.335–5.847	0.01	2	0.835–4.803	0.12
Vascular invasion	1 / 0	39 / 8	2.56	0.890–7.347	0.08			

## Discussion

The main findings of the present study were as follows: 1) patients with higher preoperative sIL-2R levels showed a worse response to IFN-alpha and sequential VEGF-targeting therapy, and multivariate analysis demonstrated that preoperative elevation of sIL-2R was an independent prognostic factor for shorter overall survival. 2) The serum level of sIL-2R largely paralleled the response to therapy over time. 3) Preoperative serum sIL-2R displayed a positive correlation with preoperative serum soluble PD-L1 and with expression of pAkt(Ser-473) by the primary tumor. Since blood samples are easier to obtain than tissue samples, blood biomarkers are preferable for assessing tumor progression and the response to therapy, as well as for personalized treatment. Our findings suggest that sIL-2R could possibly be employed to assess the biological behavior and progression of ccRCC, as well as to predict the response to IFN-alpha combined with sequential VEGF-targeting therapy.

IL-2R signaling has an important role in tolerance and in the immune response [9–11]. Tregs are a subset of CD4+ T cells that constitutively express CD25 (alpha-chain of the IL-2R), and are involved in immunoregulation [7]. Serum sIL-2R and the number of CD4+ Tregs were reported to display a positive correlation in cancer patients [19]. Tumors express numerous antigens, including self-antigens. Tregs are essential for suppression of T cell responses to tumor-associated antigens and for maintaining tolerance to self-antigens [7].

IL-2 and IL-2R are involved in immune responses by inducing the PI3K/Akt/mTOR pathway [9–11], and this pathway is highly activated in RCC [12]. Inhibition of Akt blocks transcription of glucose transporter protein-1 (GLUT1) and its translocation to the plasma membrane, where it promotes glucose utilization independently of any proliferative effect [20]. Increased glucose uptake, mainly mediated by GLUT-1, is associated with the increased dependence of tumor cells on glycolysis in the presence of oxygen (Warburg effect), and such reprogramming of cellular metabolism is considered to be a “hallmark of cancer” [2]. RCC demonstrated a shift of metabolism towards aerobic glycolysis due to impaired oxidative phosphorylation [21]. There are two forms of mTOR, mTOR complex 1 (mTORC1) and mTOR complex 2 (mTORC2), which have different intracellular functions. mTORC1-pS6 signaling promotes translation and protein synthesis, while mTORC2-pAkt(Ser-473) signaling influences energy metabolism and cell survival [22], and it has a very important role in RCC [23]. We recently reported that elevated pAkt(Ser-473) expression by the primary tumor shows a correlation with the invasiveness and metastatic potential of RCC, as well as with an unfavorable prognosis [18]. We have also previously demonstrated that higher expression of pAkt(Ser-473) in the primary tumor leads to a worse response of metastases to treatment with IFN-alpha plus low-dose sorafenib [15]. In addition, Jonasch et al. reported that detection of increased pAkt(Ser-473) expression by microarray analysis was related to worse survival after treatment with IFN-alpha plus sorafenib [24]. In the present study, the preoperative serum sIL-2R level was positively correlated with pAkt(Ser-473) expression by the primary tumor, and patients with higher preoperative sIL-2R levels and higher pAkt(Ser-473) expression showed a poor response to IFN-alpha with sequential VEGF-targeting therapy. Although we did not investigate the relationship of sIL-2R to pAkt(Ser-473) or the direct role of sIL-2R in tumor progression, our findings suggested that the serum sIL-2R level may reflect the biological aggressiveness of RCC. Accordingly, the role of sIL-2R in ccRCC should be investigated further in the future.

sIL-2R is generated by proteolytic cleavage and extracellular release of the membrane-bound form of IL-2R alpha [9–11]. sIL-2R release is associated with T cell activation and seems to be important for regulation of immunity in various settings, including infections, transplantation rejection, autoimmune inflammatory states, and cancer [9–11]. sIL-2R is reported to be produced by tumor cells and sIL-2R levels are increased in non-Hodgkin’s lymphoma, but the reasons why elevation of sIL-2R influences the prognosis are unclear. It has been suggested that sIL-2R suppresses IL-2R signaling and activates Tregs to promote tolerance of malignancy by the host immune system, leading to a poor prognosis [9–11]. Tregs promote immunosuppression and tolerance to tumor antigens in cancer patients, and these cells play the same role for microbial antigens in chronic infection [7]. An increase of circulating Tregs was reported in patients with RCC [25]. IL-2/IL-2R signaling has contradictory immunomodulatory effects, since it not only facilitates proliferation of cytotoxic CD8 T cells that kill cancer cells, but also suppresses the immune response by promoting inhibitory CD4^+^ Treg cells [9–11]. Accordingly, it can be suggested that the immunosuppressive state arising due to increased generation of Tregs may be associated with or reflected by abnormal elevation of sIL-2R.

Before metastasis occurs, cells originating from the bone marrow are recruited to the lungs, where these cells form clusters that facilitate adherence and proliferation of circulating tumor cells [26]. These marrow-derived cells produce matrix metalloproteinase (MMP)-9, which may promote invasion by cancer cells [27]. Yoshida et al. reported that serum sIL-2R and the number of CD68-positive macrophages in the tumor microenvironment were positively correlated, and functional studies performed in lymphoma have shown that MMP-9 is largely produced by tumor-associated macrophages (TAMs) and plays an important role in facilitating sIL-2R production [28]. Myeloid-derived suppressor cells, which phenotypically resemble partially differentiated granulocyte-macrophages and myeloid precursors of the monocytic lineage, are dramatically increased in the circulation of tumor-bearing animals and patients with cancer. Under certain experimental conditions, these progenitor cells undergo differentiation into antigen-presenting cells (APCs), including dendritic cells and macrophages [29]. TAMs are the major inflammatory cell population in tumors and orchestrate various processes, such as the diversion and twisting of adaptive responses, tumor vessel growth, tumor cell proliferation, deposition and remodeling of intercellular matrix, and creation of a metastatic niche with subsequent metastasis, as well as influencing the response to hormones or chemotherapy [30]. MMP-9 is required for production of sIL-2R and TAMs are the main source of MMP-9. Since myeloid-derived suppressor cells, especially TAMs, promote tumor growth by acting in the local tumor microenvironment, it seems likely that TAMs, MMP9, and sIL-2R all play a role in establishing an immunosuppressive environment that facilitates tumor progression. Tumors express a large variety of antigens, which include self-antigens. Tumors are infiltrated by Tregs and myeloid-derived suppressor cells that block local T cell responses through direct cell-cell contact [7, 29]. Accordingly, the relation of sIL-2R to Tregs and myeloid-derived suppressor cells circulating in the blood or in tumor tissues should be investigated in patients with RCC.

Cancer immunoediting allows an immunologically sculpted tumor to begin to grow progressively in the escape phase until the lesion becomes clinically apparent and establishes an immunosuppressive microenvironment, and immunoediting also promotes tumor growth in which poorly immunogenic and immunoevasive transformed cells are key players along with CD8+ T cells, CD4+ Tregs, and PD-L1 [1]. Both preclinical and clinical studies have demonstrated that suppressing the PD-1/PD-L1 pathway leads to augmentation of antitumor activity, partly by increasing the CD4+ Teff–Treg ratio within tumors [31]. Therefore, targeting T cells with anti-PD-1/PD-L1 antibodies may possibly overcome the escape mechanisms employed by malignancies and restore the equilibrium of the immune system or even facilitate tumor destruction [3, 4]. Recent studies have shown that active glycolysis in tumor cells depletes extracellular glucose and restricts its availability to host cells, leading to impairment of T cell glycolytic metabolism, while the expression of PD-L1 by tumor cells promotes constitutive activation of the Akt/mTOR pathway and treatment with anti-PD-L1 antibodies attenuates both glycolysis and phosphorylation of Akt [13, 14]. Therefore, our finding that sIL-2R is associated with PD-L1 and pAkt(Ser-473) has implications in relation to tumor biology and host-tumor interactions, suggesting that it may be worthwhile to determine the molecular mechanisms through which sIL-2R, PD-L1, and pAkt act cooperatively or independently in the tumor microenvironment.

This study had several limitations, including its retrospective design, investigation of a relatively small patient population, and a short follow-up period. However, we showed that an elevated preoperative serum level of sIL-2R was an independent prognostic factor for poor overall survival. The preoperative sIL-2R level was positively correlated with the preoperative serum level of soluble PD-L1 and with expression of pAkt(Ser-473), which has a role in progression of RCC, by the primary tumor. Furthermore, we found that the serum sIL-2R level remained nearly constant when RCC showed a good response to IFN-alpha with sequential VEGF-targeting therapy, while sIL-2R began to increase when resistance to therapy developed, indicating that monitoring serum sIL-2R may help to assessing tumor activity (at least in patients with ccRCC). Thus, our clinical observations suggested that serum sIL-2R could be used as a marker for the status of RCC, but the exact role of sIL-2R has yet to be elucidated, including how and why it is generated, the implications of an increase of sIL-2R, and how sIL-2R cooperates with other immune players. Our findings require further validation by a prospective study, preferably a larger-scale prospective controlled clinical trial. Although it is not easy to correctly assess the status of a patient with currently available methods, serial measurement of the serum level of sIL-2R may provide guidance about the current disease status. Compared with serial biopsy, a blood test is preferable as a biomarker for assessing tumor behavior and the host immune response during anticancer therapy. To confirm the clinical utility of sIL-2R with a high level of evidence, a prospective study should be performed to demonstrate that this potential biomarker can be used as the basis for clinical decisions that improve patient outcomes.

## Conclusions

sIL-2R has a role in T cell activation and regulation of the immune response. In patients with RCC, preoperative elevation of sIL-2R was associated with a higher serum level of PD-L1 and increased expression of phosphorylated Akt in the primary tumor, as well as a worse response to IFN-alpha and sequential VEGF-targeting therapy. Elevation of sIL-2R was also demonstrated to be an independent prognostic indicator of shorter overall survival. Furthermore, the serum level of sIL-2R largely paralleled the response to therapy over time. Our findings suggest that monitoring serum sIL-2R might facilitate assessment of the biological aggressiveness and progression of RCC, as well as helping to predict the effectiveness of treatment. Although the mechanism underlying elevation of sIL-2R in RCC is still unclear, further investigation is warranted to delineate the usefulness of sIL-2R for assessing disease progression and the response to therapy, as well as for designing personalized treatment.

## Abbreviations

Akt: Protein kinase B
APC: Antigen-presenting cells
ccRCC: Clear cell renal cell carcinoma
CD: Cluster of differentiation
CR: Complete response
CT: Computed tomography
ECOG: Eastern Cooperative Oncology Group
EU: European Union
Foxp3: Forkhead box P3
GLUT1: Glucose transporter protein-1
HEP: Liver
HIF: Hypoxia-inducible factor
IL: Interleukin
IL-2R: Interleukin-2 receptor
JAK: Janus kinase
KPS: Karnofsky performance status
LYM: Lymph node
MAPK: Mitogen-activated protein kinase
MMP: Matrix metalloproteinase
MRI: Magnetic resonance imaging
MSKCC: Memorial Slone-Kettering Cancer Center
mTOR: Mammalian target of rapamycin
mTORC1: mTOR-raptor complex
mTORC2: mTOR-rictor complex
OS: Overall survival time
OSS: Bone
pAkt: Phosphorylated-Akt
PD: Progressive disease
PD-1: Programmed cell death 1
PD-L1: Programmed cell death 1-ligand 1
PI3K: Phosphatidylinositol 3‘kinase
PLE: Pleura
pN: Pathological Nodes
PR: Partial response
PS: Performance status
pT: Pathological Tumor
PUL: Lung
RCC: Renal cell carcinoma
RECIST: Response Evaluation Criteria in Solid Tumors
S.D.: Standard deviation
SD: Stable disease
Ser: Serine
sIL-2R: Soluble interleukin-2 receptor
STAT: Signal transducer and activator of transcription
TAM: Tumor-associated macrophages
Teff: Effector T cell
TILs: Tumor-infiltrating T lymphocytes
Treg: Regulatory T cells
USA: United States of America
VEGF: Vascular endothelial growth factor
